# “Best practices in risk reducing bilateral salpingo-oophorectomy: the influence of surgical specialty”

**DOI:** 10.1186/s12957-017-1282-5

**Published:** 2017-12-11

**Authors:** Dominique R. Malacarne, Leslie R. Boyd, Yang Long, Stephanie V. Blank

**Affiliations:** Department of Obstetrics and Gynecology, New York University School of Medicine, 462 1st Avenue, Rm 9 E2, New York, NY 10016 USA

**Keywords:** Best practices, BRCA, BSO, Optimal prophylaxis, Risk reduction

## Abstract

**Background:**

Risk-reducing bilateral salpingo-oophorectomy (RRBSO) increases survival in patients at high risk of developing ovarian cancer. While many general gynecologists perform this procedure, some argue it should be performed exclusively by specialists. In this retrospective observational study, we identified how often optimal techniques were used and whether surgeons’ training impacted implementation.

**Methods:**

We used the ACOG guidelines highlighting various aspects of the procedure to determine which elements were consistent with best practices to maximize surgical prophylaxis. All cases of RRBSO from 2006 to 2010 were identified. We abstracted data from the operative and pathology reports to review the techniques employed. Fisher’s exact test and chi-square were utilized to compare differences between groups (InStat, La Jolla, CA).

**Results:**

Among 263 RRBSOs, 22 were performed by general gynecologists and 241 by gynecologic oncologists. Gynecologic oncologists were more likely to perform pelvic washings—217/241 vs. 10/22 (*p* < .0001). They were more likely to include a description of the upper abdomen—220/241 vs. 12/22 (*p* < .0001). Oncologists were more likely to utilize a retroperitoneal approach to skeletonize the infundibulopelvic ligaments—157/241 vs. 3/22 (*p* < .0001). When operations were performed by oncologists, the specimens were more often completely sectioned—217/241 vs. 16/22 (*p* = .003). The use of a retroperitoneal approach among gynecologic oncologists increased over the study period (chi-square for trend, *p* < .0001). There was no visible trend in performance improvement in any other area when looking at either group.

**Conclusion:**

Gynecologic oncologists are more likely to adhere to best practice techniques when performing RRBSO, though there was room for improvement for both groups.

## Background

Women carrying a deleterious mutation in the BRCA 1 or BRCA 2 genes have an elevated risk of developing ovarian, fallopian tube, and breast malignancies in their lifetimes [1–3]. While breast cancer screening has been shown to result in early detection of breast cancer, there is no effective, non-invasive screening strategy to detect ovarian or tubal cancer and thereby decrease the cancer incidence in this high-risk group. Though extreme, surgical prophylaxis after completion of childbearing is a recommended strategy for decreasing cancer risk in these women [4]. Risk-reducing bilateral salpingo-oophorectomy (RRBSO) has been shown to reduce the risk of ovarian cancer by over 80% in women with a BRCA 1 or BRCA 2 mutation [5–7]. RRBSO is the most effective means of decreasing the incidence of carcinoma as well as mortality associated with this malignancy in this high-risk population [8, 9].

Optimal techniques for RRBSO have been defined [10–15]. The Society of Gynecologic Oncology recommends obtaining peritoneal washings, surveying the entire abdomen, entering the retroperitoneal space to ensure removal of all adnexal tissue, removing fallopian tubes at their uterine insertion point, and serially sectioning all specimens during pathological evaluation [4]. Similarly, ACOG recommends that “Risk-reducing salpingo-oophorectomy … should include careful inspection of the peritoneal cavity, pelvic washings, removal of the fallopian tubes, and ligation of the ovarian vessels at the pelvic brim. If hysterectomy is not performed, care must be taken to completely remove the fallopian tubes to the level of the cornu.” ACOG and Society of Gynecologic Oncology (SGO) similarly recommend that adnexal specimens be sectioned in 2–3 mm sections, for pathologic examination [4, 16]. These systematic methods have been shown to increase detection of early neoplastic changes and occult malignancy at the time of risk-reducing surgery [10, 14, 17]. The reported rates for detection of occult malignancy vary widely, from 0.6 to 18.5% [18–23], and it is possible that this variation is related to differences in surgical technique.

At our institution, we care for a large population of women with a genetic predisposition to ovarian cancer, many of whom choose to undergo RRBSO. Both general gynecologists and gynecologic oncologists perform these procedures. We hypothesized that, because of their specialized training, gynecologic oncologists are more likely to adhere to practices that provide optimal prophylaxis for patients undergoing RRBSO. In this study, we evaluated how often ideal practices were followed and if the surgeon’s training was related to implementation.

## Methods

The study was reviewed by and run under the auspices of the NYU School of Medicine Institutional Review Board. All cases of RRBSO performed from 2006 to 2010 at NYU Langone Medical Center were identified through billing records by cross-referencing patients undergoing bilateral salpingo-oophorectomy (either alone or in conjunction with hysterectomy) and those with a personal or family history of breast cancer (V10.3 or V16.3), family history of ovarian cancer (V16.41), or genetic predisposition to ovarian or breast cancer (V84.02 or V84.01). All surgeries were verified using both surgical and pathological reports. A patient record was eligible for inclusion if RRBSO had been performed. Patients were included if there was either a documented mutation in BRCA1 or BRCA2 or a strong family history of breast and/or ovarian cancer as documented by the operating surgeon or a genetic counselor. In our population, a “strong family history” was determined based on whether patients met the standard criteria for BRCA testing. Patients with known gynecological cancer, suspected gynecologic pathology as the indication for their surgery (e.g., abnormal ultrasound), or those without complete operative reports were excluded.

The type of surgeon (gynecologic oncologist vs. general gynecologist) and date of procedure were recorded. Operative reports were examined for the following documentation, based on best practice guidelines for RRBSO as stated in ACOG Practice Bulletin #89: were pelvic washings performed; was the upper abdomen inspected; were the peritoneal surfaces inspected; and was a retroperitoneal approach used to skeletonize the infundibulopelvic (IP) ligament [4, 16]. The pathology report for each patient was reviewed to determine if the applicable preoperative diagnosis was noted and if proper processing occurred. Since November 2005, the gynecologic pathology department at our institution has followed a standardized protocol for all adnexal specimens sent as risk-reducing surgical specimens. These include serial evaluation of the entire ovary and fallopian tubes with sectioning at 2–3 mm intervals, as compared to representative sections which are the practice when risk-reducing processing is not indicated. Fisher’s exact test and chi-squared tests were utilized to compare differences between surgeries performed by general gynecologists vs. gynecologic oncologists (InStat, La Jolla, CA). *p* values less than 0.05 were considered statistically significant.

## Results

Two hundred ninety patients were originally identified for eligibility, of which 263 were evaluable. Twenty-seven patients were excluded from further study due to incorrect identification of surgery type (25) or incomplete medical record (2). BRCA mutation status was documented for each case, and the demographic characteristics of each group are shown in Table 1.

**Table 1:** Demographics by surgeon group

Demographic data	General gynecologist (*n* = 22)	Gynecologic oncologist (*n* = 241)	*p* value
Age (mean, SD)	51.6 (8.78)	49.1 (8.93)	0.2
Parity (mean, SD)	1.45(1.36)	1.31(1.29)	0.6
BMI (mean, SD)	26.1 (5.83)	25.9 (5.31)	0.8
Personal Hx of Breast CA (*n*, %)	14 (64)	179 (74)	0.3
1st degree relative with breast CA (*n*, %)	10 (45)	106 (44)	0.9
Known genetic mutation (*n*, %)	9 (41)	104 (43)	0.9
BRCA mutation status			
BRCA1+ (*n*, %)	1 (4)	47 (20)	0.09
BRCA2+ (*n*, %)	3 (14)	36 (15)	1.0
BRCA+, type not specified (*n*, %)	2 (9)	9 (4)	0.23
BRCA negative (*n*, %)	0	1 (1)	1.0
BRCA unknown (*n*, %)	16 (73)	148 (60)	0.3

Among 263 RRBSOs, 22 were performed by general gynecologists and 241 by gynecologic oncologists. Gynecologic oncologists were more likely to perform pelvic washings 217/241 (90%) when compared to generalists 10/22 (45%, *p* < .0001). They were also more likely to include a description of the upper abdomen in the operative report 220/241 (91%) vs. 12/22 of general gynecologists (55%, *p* < .0001). Oncologists were more likely to utilize a retroperitoneal approach to skeletonize the IP ligaments 157/241 (65%) when compared to generalists (3/22, 14%, *p* < .0001).

**Fig. 1:**
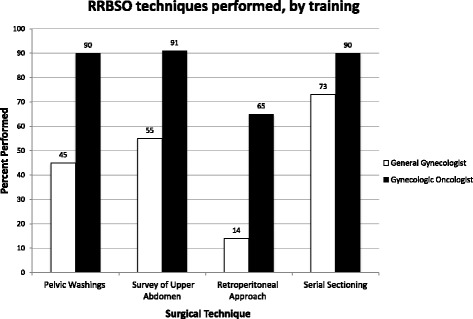
Percentage of RRBSO techniques performed, by level of training

**Fig. 2:**
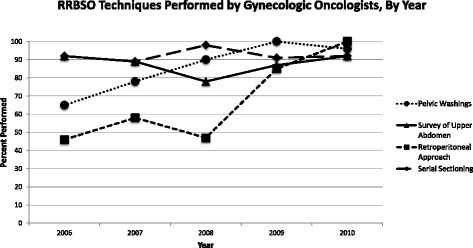
The percentage of each technique used by gynecologic oncologists when performing RRBSO, by year

**Fig. 3:**
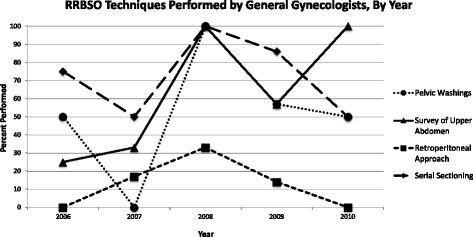
The percentage of each technique used by general gynecologist when performing RRBSO, by year

When surgeries were performed by oncologists, the ovaries and fallopian tubes were more likely to be completely sectioned—217/241 (90%) vs. 16/22 (73%, *p* = .003, Fig. 1). The use of a retroperitoneal approach among gynecologic oncologists increased over the study period when analyzed by year. 12/26, 44/76, 27/59, 47/55, and 25/25 cases were thus performed in the years 2006 through 2010, respectively (chi-square for trend, *p* < .0001, Fig. 2). There was no visible trend in the performance of these parameters over time when evaluating the general gynecologist group (Fig. 3).

**Fig. 4:**
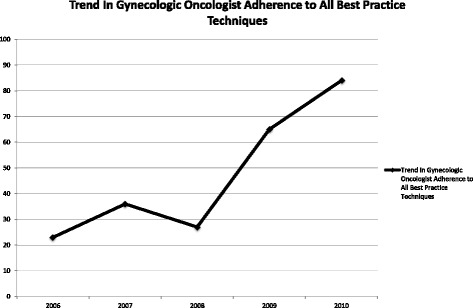
Trend in gynecologic oncologist adherence to all best practice techniques

Additionally, we analyzed how often each surgeon group adhered to best practices globally. Only 2 of the 22 procedures (9%) performed by generalists were found to comply with all suggested RRBSO techniques. In comparison, 41% of all RRBSOs performed by gynecologic oncologists had complete adherence, and the trend toward complete adherence was statistically significant when analyzed by year (*p* < .0004, Fig. 4).

Seven patients had occult malignancy noted on the final pathology, all of whom had surgery performed by gynecologic oncologists. This represents a 2.6% incidence of occult carcinoma in our prophylactic salpingo-oophorectomy specimens. Table 2 lists surgery characteristics for each of these patients, as well as final pathology and prognosis. Three patients possessed a deleterious BRCA mutation (two with BRCA 1, one patient with BRCA 2). All three of these patients were diagnosed with primary fallopian tube cancer at the time of RRBSO. All three of these patients underwent an additional staging operation, followed by chemotherapy. Of the four patients with unknown mutation status, metastatic breast cancer was the diagnosis on final specimen pathology in three instances. Two of these patients had already been diagnosed with metastatic disease (either to lymphatics or bone). Lastly, one patient was diagnosed with a Leydig cell tumor of the ovary. All seven patients were diagnosed based on pathology specimens. No pelvic washings that were submitted were found to be positive for malignancy. Patients were followed through 2017. Of the three patients with diagnoses of fallopian tube cancer at the time of RRBSO, all have been free of malignancy since the time of diagnosis. There have been no known deaths. Two of the three patients with metastatic disease went on to have further progression of disease (Table 2).

**Table 2:** Characteristics of patients diagnosed with occult malignancy

Patient	Mutation status	Age	Diagnosis	Prognosis
1	Unknown	45	Metastatic breast cancer to ovary (dx at RRBSO 2007)	Patient last seen 2011: diagnosis right femoral head metastases 2010 s/p XRT
2	Unknown	54	Leydig cell tumor of the ovary	Patient last seen 2012: no evidence of disease
3	Unknown	43	Metastatic breast cancer to ovary (known metastases to lymph nodes)	Lost to follow-up
4	Unknown	45	Metastatic breast cancer to ovary (known metastases to bone)	2010: new bone metastases; 2012: extensive bone mets, vertebral body compression fractures; 2017: stable lesions, on denosumab
5	BRCA1+	44	Papillary serous carcinoma of the fallopian tube	s/p 3 cycles carboplatin; no evidence of disease since 2009
6	BRCA+	44	Papillary serous carcinoma of the fallopian tube	s/p 6 cycles carboplatin/Taxol; no evidence of disease since 2009
7	BRCA2+	68	Papillary serous carcinoma of the fallopian tube	s/p carboplatin/Taxol × 3 cycles; no evidence of disease since 2007

## Discussion

At our institution, having specialty training was more likely to be associated with adherence to ideal practices for risk-reducing bilateral salpingo-oophorectomy (BSO). Further, practice improvement over time was noted for those cases performed by gynecologic oncologists, although ideal compliance remained around 40% for this group. Alternatively, there was no similar trend when analyzing the generalists’ surgical practices. This finding is likely secondary to the increased awareness of the surgical issues pertinent to these cases among gynecologic oncologists. This also highlights the importance of further awareness and proper training among all providers performing RRBSO. In many parts of the world, specialists may not be accessible, and proper training of generalists is imperative if correctly performed RRBSO is to be widely accessible.

At our hospital a particular effort was made to formalize practices for pathologists sectioning these specimens; however, no formal training was instituted for the surgeons. Some of the aspects required to conform to ideal practices do not require specific surgical technique, e.g., correct documentation on the pathology request to ensure use of the “risk-reducing protocol” for specimen sectioning. Prior to performing these procedures, the pathologist should be consulted. If the required sectioning cannot be instituted, patients need to be referred to alternative institutions where this practice is standard, as new evidence further supports that this technique increases diagnosis of microscopic malignancy, and may even aid in the identification of premalignant conditions in certain populations [24, 25]. Other aspects, such as ensuring that the infundibulopelvic ligament is dissected a sufficient distance from the ovary, may be challenging. Although ACOG does not recommend a retroperitoneal approach specifically, this may, in fact, be the safest way to confirm transection of the IP ligament at the pelvic brim. In these situations, patients should be referred appropriately. RRBSO should only be performed under circumstances in which compliance with all elements of best practice can be expected preoperatively.

The use of peritoneal lavage at the time of prophylactic surgery for high-risk women has been previously reviewed [10, 11, 21, 22]. Now that we implement close sectioning of the ovaries and fallopian tubes, cytology may play a less important role. We found it was uninformative in our small sample.

There are several limitations to this study. This is a single-institution, retrospective study, and therefore, its applicability to other institutions is unknown. Although we evaluated over 250 cases, the cases performed by general gynecologists made up a relatively small number. Additionally, we do not have consistent follow-up data on patients to determine the incidence of post-BSO primary peritoneal cancer and therefore cannot comment as to whether lack of adherence to best practices resulted in worse outcomes. Despite this, we believe there are clear trends shown in our data which warrant further investigation.

## Conclusions

In conclusion, we found that gynecologic oncologists were more likely to adhere to ideal practices for risk-reducing BSO. Guidelines published by both ACOG and SGO outline similar best practice techniques and acknowledge that this procedure can be performed by either generalists or gynecologic oncologists. In many geographic areas, patients will have better access to general gynecologists [26]. Greater efforts to educate providers about best practices may be warranted. Dedicated education on RRBSO techniques could be implemented to optimize the performance of this important and potentially life-saving procedure by all practitioners that offer it.

## Abbreviations

BSO: Bilateral salpingo-oophorectomy
RRBSO: Risk-reducing bilateral salpingo-oophorectomy
